# 

**Published:** 1887-10

**Authors:** 


### AMERICAN PUBLIC HEALTH ASSOCIATION.

### SIXTEENTH ANNUAL SESSION.

The Local Committee of Arrangements announce that the following arrangements have been made preliminary to the meeting of "The American Public Health Association," to be held in Memphis, November 8th—11th, 1887.

The meetings of the Association will be held in the Young Men's Hebrew Association Hall, corner of Second and Union Streets, commencing at 10 a. m., November 8th.

All Railroads in the Southern Passenger Association, the Central Traffic Association, and the Western States Association, have authorized one and one-third rates. That is one full rate coming and one-third rate returning on certificate of Secretary.

The Trunk Lines Association which embraces New England States and Canada yet to be heard from, but the Committee is induced to believe it will authorize same rates.

The following rates are given by the Hotels:

The GAYOSO HOTEL, Shelby Street, $2.50 and $3.00 per day.

The PEABODY HOTEL, corner of Main and Monroe Streets, $2.50 and $3.00 per day.

GASTON'S HOTEL, South Court Street, European Plan, $1.00 per day, for room, Restaurant in Hotel.

DUFFY'S HOTEL, corner Main and Adams Streets, European and American Plan. Rooms 50c., 75c. and $1.00. Restaurant in Hotel. American Plan $2.00 per day.

Rooms will be engaged at the Gayoso for the President, Secretary and Treasurer of the Association.

HON. ROBERT L. TAYLOR, Governor of Tennessee, will deliver an address on the evening of the 8th.

The Local Committee hopes and expects a full representation of members of the Association at this meeting, as well as a large accession of new members, and will spare no effort to make their sojourn in Memphis both pleasant and profitable.

G. B. THORNTON, M.D., Chm. of Local Com. of Arrangements.
D. T, PORTER, Chairman of Finance Committee.
R. W. MITCHELL, M. D., Chairman of Reception Committee.
Major M. BURKE, Chairman of Transportation Committee.
F. L. SIM, M. D., Chm. of Com. on Printing and Publication.

The next session of the "East Texas Medical Association" will be held at Jacksonville, Texas, on 1st Tuesday in January.

That of the "Austin District Medical Society" will be held at Austin, December, 2nd Thursday.—Dr. F. T. Paine, of Comanche, is expected to be present by invitation, and to read a paper, and to make some experiments and demonstrations with electricity in the treatment of chronic disease.

